# Role for Positive Schizotypy and Hallucination Proneness in Semantic Processing

**DOI:** 10.3389/fpsyg.2020.542002

**Published:** 2020-09-02

**Authors:** Saskia de Leede-Smith, Steven Roodenrys, Lauren Horsley, Shannen Matrini, Erin Mison, Emma Barkus

**Affiliations:** ^1^School of Psychology, University of Wollongong, Wollongong, NSW, Australia; ^2^Cognitive Basis of Atypical Behaviour Initiative (CBABi), School of Psychology, University of Wollongong, Wollongong, NSW, Australia; ^3^School of Psychology, Northumbria University, Newcastle upon Tyne, United Kingdom

**Keywords:** schizotypy, hallucinatory proneness, semantic processing, semantic priming, positive schizotypy

## Abstract

Semantic processing underpins the organization of verbal information for both storage and retrieval. Deficits in semantic processing are associated with both the risk for and symptoms presented in schizophrenia. However, studies are mixed and could reflect the confounding effects of medication and symptom heterogeneity. Therefore, we considered whether two risk phenotypes, positive schizotypy and hallucinatory predisposition, present in the general population were associated with differential responding profiles for a semantic processing task. One hundred and eighty-three participants completed the Schizotypal Personality Questionnaire, Launay-Slade Hallucination Scale, National Adult Reading Test, a handedness measure, and a computerized semantic relatedness judgment task. Pairs of words were related through their dominant or subordinate meanings, or unrelated. Participants were divided into four groups using a mean split on cognitive-perceptual (positive) schizotypy and hallucination proneness. Significant differences between groups were found for reaction time on the semantic relatedness task, with the high cognitive-perceptual schizotypy groups responding significantly slower to all word pairs compared to their low scoring counterparts. There was some evidence that high hallucination proneness was associated with significantly faster reaction times which may reflect disinhibitive processes, however additional support is required. The results suggest that these two components of psychosis risk are associated with different patterns of responding to semantic processing. More diffuse activation of semantic information appeared to be associated with positive schizotypy, while those predisposed to hallucinations appeared to respond quicker. These results have significant implications in the re-conceptualization of hallucination proneness as distinct from positive schizotypy. Additional research is required to investigate the association between psychotic-like experiences separate from personality variables such as positive schizotypy and semantic processing.

## Introduction

Semantic processing is the cognitive consideration of word meanings, where review of a particular word automatically stimulates activation for other words with similar and related meanings. Semantic processing abnormalities are a central feature of schizophrenia (Minzenberg et al., 2002; Sekulic Sovic et al., 2019). Patients with schizophrenia exhibit semantic processing abnormalities (Langdon et al., 2002; Surguladze et al., 2002; Brebion et al., 2004; Langdon and Coltheart, 2004). In patients with schizophrenia, semantic processing deficits may be the result of a reduced ability to integrate context with meaning (Iakimova et al., 2005) or disorganization of the mechanisms necessary to facilitate activation across the semantic network (Kumar and Debruille, 2004; Soriano et al., 2008; Niznikiewicz et al., 2010). However, there are confounds in collecting data in patients with schizophrenia including medication, substance use, the effects of diagnosis and hospitalization, and chronicity of symptoms. Therefore one approach to providing more enriched information for risk factors for psychosis is to consider schizotypy, an analog or proxy for symptoms in patients captured in the general population.

With schizophrenia at the extreme end, schizotypy exists along a continuum of psychosis (Kaymaz and van Os, 2010; Tabak and de Mamani, 2013), with at risk mental state and schizotypal personality disorder being intermediaries between general population high schizotypes and diagnosed psychotic disorders (Debbané et al., 2014; Debbané and Mohr, 2015). Although research linking community samples assessed on schizotypy to diagnosable relevant disorders is still in its infancy, there is evidence that schizotypy is a useful risk indicator for future psychosis (Debbané et al., 2014; Zarogianni et al., 2017). Psychometrically defined schizotypy is associated with the future development of positive and negative psychotic symptoms, substance use and affective symptoms as well as psychotic disorders (Blanchard et al., 2011; Kwapil et al., 2013; Racioppi et al., 2018). Schizotypy is a normally distributed multidimensional personality trait resembling dispositional features of schizophrenia. Defining features of schizotypy include cognitive-perceptual experiences, interpersonal characteristics, and disorganization of thinking and behavior. Females tend to score higher on the positive dimensions of schizotypy, while males report higher scores on the negative and disorganized features (Fonseca-Pedrero et al., 2018; Mimarakis et al., 2018), although sex differences in schizotypy are considered small (Barron et al., 2018). There may also be sex differences in the cognitive, psychological and behavioral correlates of schizotypy, with associations with shizotypy being significant in females rather than males in some studies (Deptula and Bedwell, 2015; Mitchell et al., 2015; Toutountzidis et al., 2018). However, not all studies find sex differences for psychological or behavioral correlates of schizotypy (Rossell et al., 2014; Rössler et al., 2015). Schizotypy can be understood as a biological and cognitive vulnerability to psychosis (MacDonald et al., 2001; Walter et al., 2016; Kemp et al., 2020). Abnormalities in the lateralization of language processing are associated with schizotypy (Kravetz et al., 1998; Weinstein and Graves, 2002; Mohr et al., 2005; Suzuki and Usher, 2009), and positive schizotypy specifically (Nunn and Peters, 2001; Hori et al., 2008). Therefore, additional consideration needs to be given to variation in semantic processing attributable to positive schizotypy and other psychotic-like experiences.

Semantic priming is one of the main tasks used to investigate semantic networks. A priming word is presented (e.g., ball), followed by a target word which is semantically related (e.g., soccer) or unrelated (e.g., coffee). Semantic priming occurs when the participant responds to the related word significantly faster/more accurately than the unrelated word. The facilitation for the related word occurs because activation spreads across links in the network and semantically related nodes are located closer together, whilst unrelated words are further apart. Indirect priming occurs when the prime and target are not directly related, but mediated by another concept (e.g., prime CAT and target CHEESE are mediated by MOUSE). Indirect priming reflects a less constrained, or more diffuse, spread of activation (Weisbrod et al., 1998; Zeev-Wolf et al., 2014, 2015). Greater indirect priming is associated with positive schizotypy (Kerns and Berenbaum, 2000; Morgan et al., 2006), or disorganized schizotypy (Tan and Rossell, 2017), and diffuse (i.e., less targeted or disinhibited) semantic processing is associated with higher schizotypy in general (Morgan et al., 2006; Grimshaw et al., 2010). However, these findings are not consistent (Fisher and Weinman, 1989; Moritz et al., 1999; Morgan et al., 2009). Therefore, clarifying the nature of any differences in semantic processing in positive schizotypy is an aim of the present study.

Positive schizotypy is a complex trait comprising unusual beliefs, thoughts and perceptual experiences. Schizotypy is a trait that is relatively stable over time and reflects a complex component of personality, underpinned by an interaction between environment and genetic predisposition (Jang et al., 2005; Lin et al., 2007). Unusual perceptual experiences and more fully formed auditory verbal hallucinations (AVH) are captured within positive schizotypy. Unusual perceptual experiences are heterogeneous, with differential relationships with schizotypy and other psychological symptoms (Unterrassner et al., 2017). While other components of positive schizotypy remain stable over adolescence, unusual perceptual experiences decline (Debbané et al., 2013). Indeed, psychotic-like experiences in adulthood, but not adolescence, are genetically linked with psychotic and mood disorders (Barkhuizen et al., 2020). Hallucinations and schizotypy are identified as separate factors in middle-aged adults (Rössler et al., 2013). Taken together, these findings suggest that while positive schizotypy is trait-like, AVH are separable and are more transient their expression. This creates a complex scenario where individuals may differ in their predisposition to AVH but their experiencing of AVH will vary over time. AVH are experienced by 5–28% of healthy individuals at some point in their lives (Johns, 2005; de Leede-Smith and Barkus, 2013). The differences between clinical and non-clinical voice hearers have been considered. While the perceptual qualities of the experiences are similar between clinical and non-clinical people voice hearers, often non-clinical voice hearers report more control and a positive interpretation and relationship with the voices they hear (Mawson et al., 2011; Baumeister et al., 2017; Powers et al., 2017). These differences suggest that other factors, such as affective style, cognitive biases and functioning, may shape the experiences voice hearers have.

One mechanism proposed for the differential processing of ambiguous relations in high schizotypes is reduced cognitive inhibition; a process regarded as central to high schizotypy and directly related to language processing ability (Beech and Claridge, 1987). Reduced cognitive inhibition is characterized by poor discrimination between relevant and unrelated “noise” factors. Diffuse activation of semantic associates in high schizotypy may be linked to an inability to inhibit unrelated features. Failure to inhibit irrelevant semantic stimuli has been associated with schizotypy (Grimshaw et al., 2010; Humphrey et al., 2010). Furthermore, high schizotypes are significantly less likely to show negative priming (Moritz et al., 2000; Steel et al., 2007), as well as having a propensity toward a liberal response bias in different tasks (Reed et al., 2008; Humphrey et al., 2010). Collectively, these findings reflect reduced cognitive inhibition, which could underlie atypical semantic processing in high positive schizotypy. The processing of semantic relations is also specifically linked to hallucinatory experiences (Vercammen and Aleman, 2008). One of the most accepted mechanisms precipitating AVH onset is a reduced ability to inhibit intrusive thoughts and memories (Badcock and Hugdahl, 2012), believed to occur due to impaired inhibition in the top-down processing system (Kompus et al., 2011). Since inhibitory dysfunctions exist in both trait positive schizotypy and the hallucinatory experiences, it might be expected that lack of inhibition associated with AVH will also impact on semantic processing. Therefore, in the current study we will consider those who score highly on positive schizotypy and AVHs.

It is hypothesized that our groups will not differ for reaction time responses to dominant meaning related word pairs, and will be characterized by significantly faster reaction times to targets related by the dominant meaning of the prime compared to unrelated targets. For subordinate meaning word pairs, it is expected that the low positive schizotypy and AVH prone groups would exhibit significantly slower reaction times compared to dominant pairs, due to the inhibition of subordinate meanings (the expected semantic function in the general population; Burgess and Simpson, 1988). Contrastingly, high positive schizotypy and AVH proneness will interact with meaning, such that subordinate meanings for target words will be activated by the prime word, due to reduced inhibitory function in these groups. Therefore, a smaller difference in reaction time between dominant and subordinate meaning primes is hypothesized for these groups. Accuracy and decision making bias is investigated using signal detection outcomes.

## Materials and Methods

### Participants

One hundred and eighty-three undergraduate students from the University of Wollongong, NSW, Australia, took part in the study [mean age 22 years (SD 7.16), age range 17–60 years, 75.4% female] and were recruited on an opportunity sample basis.

### Measures

#### Participant Characteristic Measures

Each participant completed an initial demographic questionnaire, requiring details such as age and sex. Handedness was determined, then participants completed the Schizotypal Personality Questionnaire (SPQ; Raine, 1991), Launay-Slade Hallucination Scale (LSHS; Launay and Slade, 1981), National Adult Reading Test (NART; Nelson and Willison, 1991), and finally the computerized Semantic Ambiguity Task [adapted with permission from Grimshaw et al. (2010)].

The SPQ consists of 74 items requiring either a yes or no response summed to produce a total score and three dimensions: Cognitive Perceptual (CP), Interpersonal, and, Disorganized. The overall mean score for participants on the SPQ was 27.28 (S.E. 1.18). The focus of the current study is positive schizotypy so participants were divided into groups based on their CP score. The mean score for participants on CP was 10.57 (S.E. 0.52). Those with a score above the mean were the high CP schizotypy group, while those scoring at or below the mean were the low CP schizotypy control group.

The LSHS is a 12-item questionnaire captures hallucinatory predisposition with a total score being calculated with a possible range of 0–12. The overall mean score for participants was 3.43 (S.E. 0.17). Those with a score above the mean were grouped as the high AVH prone group, whilst those scoring at or below the mean were the low AVH prone group.

Handedness was determined by the hand participants preferred to use when performing nine various tasks (i.e., writing, sweeping with a broom, unscrewing the lid of a jar). If Right/Left hand was used for 7+ activities, this determined handedness. However, if the Right/Left hand was used for less than seven activities, the individual was classified as “Mixed” handedness.

The NART was used to estimate verbal intellectual ability. Participants read aloud 50 atypically spelled words increasing in difficulty. The number of pronunciation errors was recorded.

#### Semantic Ambiguity Task

In the present study, semantic processing was evaluated via the use of homographs (words with two different meanings). Disambiguating meaning in the English language requires the activation of the appropriate semantic pathway, and deactivation (inhibition) of the incongruous alternate meaning(s). In the current relatedness judgment task [from Grimshaw et al. (2010)], participants were presented with an ambiguous word (prime), immediately followed by another word (target), which is either: related to the dominant meaning of the prime, related to the subordinate meaning of the prime, or unrelated to the prime.

This task was conducted on a laptop in a quiet room at the University of Wollongong. Participants were told they would see one word flash up on the screen (prime), followed immediately by another word (target). Once the target disappeared they were required to indicate whether both words were related (pressing key 1) or unrelated (pressing key 2). The task comprised 144 trials, participants were asked to respond as accurately and quickly as possible.

Forty different prime words were used, along with a target word related to the prime by the dominant meaning and a target related by the subordinate meaning the prime. For example, for the prime “ball,” a dominant related target would be “round,” whereas a subordinate related target would be “dancing.” Seventy-two related word pairs were used in the task (36 dominant and 36 subordinate), along with 72 completely unrelated word pairs (where a prime was pseudo-randomly paired with one dominant and one subordinate word of a different unassociated prime). Each participant saw each prime twice: paired with either a related target (half of which were dominant, half subordinate), or an unrelated target (half of which were dominant, half subordinate). Participants also saw each target on two occasions. Counterbalancing occurred, such that if a participant viewed a prime paired with a dominant and related target, they would also see the same prime paired with a subordinate and unrelated target, and vice versa. Counterbalancing also occurred across pairings with the use of two word lists. These word lists comprised the same words, however paired differently. For example, if in the first word list the related word pair was subordinate and the unrelated word pair was dominant, this would be reversed in the second word list (so the related word pair would be dominant and the unrelated word pair would be subordinate). Participants completed the task with word list 1 *or* 2, resulting in half the participant pool completing each version of the task. Details regarding the pairings words can be found in Grimshaw et al. (2010).

Each trial was preceded by a fixation mark in the center of the screen (1000 ms), followed by a centrally presented prime word (50 ms). A stimulus onset asynchrony (SOA) of 750 ms then followed, after which the target was presented for 180 ms. A SOA of 750 ms was used because this SOA is when inhibitory processes are most likely occurring (Burgess and Simpson, 1988; Atchley et al., 1999). Participants were given 3000 ms to make a response (1 for “related” or 2 for “unrelated”), after which there was a further 3000 ms inter-stimulus interval between their response and the beginning of the subsequent trial.

### Procedure

Ethical approval to commence the study was obtained by the University of Wollongong Human Research Ethics Committee. Written informed consent was obtained from participants before testing commenced. Participants were reimbursed with course credit for their time.

### Statistical Analysis

Data analysis was conducted using SPSS Version 21 (IBM Corp, 2012). Response time analyses were based on median response times for concordant (correct) responses. To control for the random effects of both participants and items a subject (*F1*) analysis and item (*F2*) analysis were run using a Repeated Measures Analysis of Variance (ANOVA).

Performance accuracy was divided into two components: sensitivity (*d′*) and criterion (*c*). Sensitivity refers to the participant’s ability to accurately discriminate between related and unrelated targets (i.e., to respond correctly). The sensitivity analysis was completed using a Repeated Measures ANOVA. Criterion is related to the decision making bias of the participant. This bias is evident under conditions of uncertainty or ambiguity, under which participants will have a propensity to respond with either a lax or conservative pattern of response. A lax pattern of response would involve responding “related” more so than “unrelated” when uncertain, whereas in a conservative pattern of response the participant would be more likely to classify uncertain targets as “unrelated.” For the criterion measure, positive values indicate a conservative decision making bias, whereas negative values are indicative of a lax decision making bias.

The Signal Detection variables were calculated using the Macmillan and Creelman (2005) criteria:

*d′* = z(hits) – z(false alarms).

*c* = -0.5 [z(Hits) + z(false alarms)].

To allow comparison of *c* across dominant and subordinate conditions, the *c* variable needs to be on the same scale, with the mean of the unrelated distribution as the zero point. To accomplish this, an arithmetic transformation was used, where *d′* for each condition was divided by 2, with *c* then added to it. Dominant and subordinate conditions could then be compared via a *t*-test. To compare *c* between CP schizotypy and AVH prone groups *c* was then converted into relative *c′*, as suggested by Macmillan and Creelman (2005). This was done by dividing *c* by *d′.* Doing this allows the difference between the groups on *d′* to be taken into account. The *c′* for high and low CP schizotypy, and high and low AVH prone groups was then compared via the use of an ANOVA.

In cases where the number of hits or false alarms was 0 or 1, an adjustment was applied to avoid infinite values. Proportions of 0 and 1 were converted using the formula 1/(2N) and 1-1/(2N), respectively, where N symbolizes the number of trials that proportion is based upon (Macmillan and Creelman, 2005). Therefore values of 0 and 1 were converted to 0.014 and 0.986, respectively. False alarms were defined as responding “related” to an unrelated item, whereas hits were defined as the correct response (response of “related” to a related item). Given that false alarms were universal across dominant and subordinate conditions (i.e., conditions are equal in their unrelatedness), they were summed across the conditions, making the false alarm rate out of 72.

## Results

### Participants Characteristics

Main effects between the CP schizotypy groups revealed significant differences between high and low CP schizotypy groups for the SPQ Cognitive-Perceptual dimension [*F*(1, 178) = 315.17; *p* < 0.001; η^2^*_*p*_* = 0.639; High CP: *M* = 16.63 (S.D. 4.6), Low CP: *M* = 5.0 (S.D. 3.09)], SPQ total score [*F*(1, 178) = 142.272; *p* < 0.001; η^2^*_*p*_* = 0.444; High CP: *M* = 39.45 (S.D. 12.48), Low CP: *M* = 16.16 (S.D. 9.55)], Interpersonal dimension [*F*(1, 178) = 57.957; *p* < 0.001; η^2^*_*p*_* = 0.246; High CP: *M* = 16.52 (S.D. 6.99), Low CP: *M* = 7.56 (S.D. 5.98)], Disorganized dimension [*F*(1, 178) = 25.761; *p* < 0.001; η^2^*_*p*_* = 0.126; High CP: *M* = 8.3 (S.D. 3.8), Low CP: *M* = 4.25 (S.D. 3.73)] and LSHS [*F*(1, 178) = 34.654; *p* < 0.001; η^2^*_*p*_* = 0.163; High CP: *M* = 4.7 (S.D. 2.26), Low CP: *M* = 2.25 (S.D. 1.63)]. No significant differences were found between the CP schizotypy groups for sex (*p* = 0.13), age (*p* = 0.339), handedness (*p* = 0.16), or verbal intelligence (*p* = 0.300).

Significant main effects were also found between high and low AVH proneness groups for SPQ total score [*F*(1, 178) = 28.198; *p* < 0.001; η^2^*_*p*_* = 0.137; High AVH: *M* = 36.07 (S.D. 14.42), Low AVH: *M* = 19.43 (S.D. 13.126)], Cognitive-Perceptual [*F*(1, 178) = 23.412; *p* < 0.001; η^2^*_*p*_* = 0.116; High AVH: *M* = 14.26 (S.D. 6.66), Low AVH: *M* = 7.25 (S.D. 5.49)], Interpersonal [*F*(1, 178) = 7.85; *p* = 0.006; η^2^*_*p*_* = 0.042; High AVH: *M* = 14.99 (S.D. 6.97), Low AVH: *M* = 9.02 (S.D. 7.59)] and Disorganized dimensions [*F*(1, 178) = 25.93; *p* < 0.001; η^2^*_*p*_* = 0.127; High AVH: *M* = 8.33 (S.D. 3.86), Low AVH: *M* = 4.27 (S.D. 3.68)] and, unsurprisingly, LSHS [*F*(1, 178) = 283.438; *p* < 0.001; η^2^*_*p*_* = 0.614; High AVH: *M* = 5.41 (S.D. 1.56), Low AVH: *M* = 1.65 (S.D. 1.09)]. No main effects were found between the AVH proneness groups for sex (*p* = 0.58), age (*p* = 0.946), or handedness (*p* = 0.47). However the high AVH proneness group scored significantly worse on the NART compared to the low AVH proneness group [*F*(1, 178) = 4.13, *p* = 0.044; η^2^*_*p*_* = 0.023; High AVH: *M* = 28.77 (S.D. 5.29), Low AVH: *M* = 27.4 (S.D. 5.22) errors].

Demographic variables for the interaction between CP schizotypy and AVH proneness are in Table 1. There were no significant differences in sex ratios, age, handedness, SPQ total or subscale scores, or the NART. However, significant group differences were found for LSHS total score [*F*(1, 178) = 4.265, *p* = 0.04]. Pairwise comparisons revealed that all four groups differed significantly from each other (all *p* < 0.05), with those high on CP schizotypy-AVH proneness scoring highest [*M* = 5.898 (S.E 0.159)], followed by the low CP schizotypy-high AVH prone individuals [*M* = 4.333 (S.E 0.234)], then the high CP schizotypy-low AVH prone participants [*M* = 2.179 (S.E 0.23)], and those with low CP schizotypy-AVH prone scoring lowest for LSHS total score [*M* = 1.426 (S.E 0.148)].

**TABLE 1:** Demographic variables for interaction between Cognitive-Perceptual (CP) schizotypy groups and Auditory-Verbal Hallucination (AVH) proneness groups.

Variable	High CP schizotypy, High AVH prone (*n* = 59)	High CP schizotypy, Low AVH prone (*n* = 28)	Low CP schizotypy, High AVH prone (*n* = 27)	Low CP schizotypy, Low AVH prone (*n* = 68)	Test statistic and *p*-value
Sex (Male: Female)	16:43	9:19	6:21	13:55	χ^2^ = 2.254, *df* = 3, NS
Age	21.98 (7.85)	20.07 (3.54)	21.26 (4.76)	23.01 (8.33)	*F*(1, 178) = 2.505, NS
Handedness (Right:Left: Mixed)	56:3:0	25:3:0	21:5:1	58:10:0	χ^2^ = 10.322, *df* = 6, NS
SPQ total score	42.75 (11.55)	32.5 (11.63)	21.48 (7.75)	14.04 (9.42)	*F*(1, 178) = 0.711, NS
Interpersonal	17.61 (6.16)	14.21 (8.13)	9.26 (4.94)	6.88 (6.25)	*F*(1, 178) = 0.245, NS
Disorganized	9.27 (3.57)	6.25 (3.52)	6.26 (3.72)	3.46 (3.45)	*F*(1, 178) = 0.036, NS
NART total score	28.59 (5.38)	26.54 (5.06)	29.15 (5.16)	27.75 (5.28)	*F*(1, 178) = 0.15, NS

*Standard deviations are shown in parentheses.*

### Semantic Ambiguity Task Response Times

#### Group Analysis (*F1*) for Reaction Time Data

Concordant response times were analyzed in a 2 (meaning) × 2 (relatedness) × 2 (CP schizotypy group) × 2 (AVH prone group) Repeated Measures ANOVA. In this design meaning and relatedness were the within subject variables, and CP schizotypy and AVH proneness were the between subject variables. All variables met the ±2 requirements for skewness and kurtosis except for the unrelated subordinate reaction time variable, which had a kurtosis value of 3.01 (SE 0.36). As a result box plot diagrams were used to identify possible outliers. One outlier was identified and removed, with the renewed kurtosis value subsequently meeting acceptable limits. Sphericity was not violated for this data therefore no corrections were required. When *post-hoc* analyses were used the *p*-value was adjusted using Bonferroni corrections. Table 2 contains the descriptive statistics for this analysis.

**TABLE 2:** Mean of the median reaction times to concordant responses in milliseconds.

Group	Meaning	Related	Unrelated	Unrelated – Related
High CP Schizotypy,	Dominant	826 (22)	1077 (26)	251 (25)
High AVH prone	Subordinate	987 (19)	1062 (28)	75 (23)
High CP Schizotypy,	Dominant	779 (18)	1089 (32)	309 (23)
Low AVH prone	Subordinate	998 (22)	1116 (35)	118 (29)
Low CP Schizotypy,	Dominant	697 (15)	990 (24)	294 (27)
High AVH prone	Subordinate	874 (19)	958 (28)	85 (29)
Low CP Schizotypy,	Dominant	770 (19)	966 (21)	196 (20)
Low AVH prone	Subordinate	936 (18)	933 (20)	−3 (20)

*Standard deviation shown in parentheses.*

#### Task Effects

Main effects of both meaning [*F*(1, 178) = 131.607, *p* < 0.001, η^2^*_*p*_* = 0.425] and relatedness [*F*(1, 178) = 86.755, *p* < 0.001, η^2^*_*p*_* = 0.328] were significant, with participants responding significantly slower to subordinate word pairs compared to dominant [Dominant: *M* = 900 ms (S.E. 15), Subordinate: *M* = 983 ms (S.E. 16)], and unrelated word pairs compared to related [Related: *M* = 858 ms (S.E. 15), Unrelated: *M* = 1024 ms (S.E. 20)]. A significant interaction effect was also found between meaning and relatedness [*F*(1, 178) = 187.529, *p* < 0.001, η^2^*_*p*_* = 0.513, Dominant-Related: *M* = 768 ms (S.E. 16), Dominant-Unrelated: *M* = 1031 ms (S.E. 20), Subordinate-Related: *M* = 949 ms (S.E. 16), Subordinate-Unrelated: *M* = 1017 ms (S.E. 22)]. This finding is reflective of meaning impacting on reaction time responses when words are related, however when words are unrelated they are not expected to differ, as they are both the same in their “unrelatedness” regardless of meaning.

#### Group Effects

A significant main effect was found for CP schizotypy group [*F*(1, 178) = 10.78, *p* = 0.001, η^2^*_*p*_* = 0.057], with the high CP schizotypy group responding slower overall compared to the low group [High CP: *M* = 992 ms (S.E. 22), Low CP: *M* = 891 ms (S.E. 22)]. No significant interactions were found between CP schizotypy and meaning (*p* = 0.055), relatedness (*p* = 0.201), or the 3-way interaction of schizotypy with relatedness and meaning (*p* = 0.478).

No main effect was found for AVH proneness (*p* = 0.633). No significant interaction effects were documented between AVH proneness and the task factors meaning (*p* = 0.137) or relatedness (*p* = 0.555), or their interaction with each other (*p* = 0.92).

No interaction was documented between CP schizotypy and AVH proneness (*p* = 0.82). However, a significant interaction was found between CP schizotypy, AVH proneness and relatedness [*F*(1, 178) = 4.06, *p* = 0.045, η^2^*_*p*_* = 0.022]. Descriptive statistics for this analysis are in Table 3. To unpack this interaction the analysis was rerun with the file split by CP schizotypy. The interaction between AVH proneness and relatedness reached trend level significance for low CP schizotypy [*F*(1, 93) = 3.69, *p* = 0.058, η^2^*_*p*_* = 0.038], but was not significant for high CP schizotypy (*p* = 0.337). This analysis was repeated with the file split by AVH proneness. Results showed that the interaction between CP schizotypy groups and relatedness was significant for low AVH proneness [*F*(1, 94) = 6.737, *p* = 0.011, η^2^*_*p*_* = 0.067], but not for high AVH proneness (*p* = 0.641). Therefore, within low AVH prone individuals, unrelated words led to larger increases in reaction time compared to related words when individuals were also high on CP schizotypy compared to low CP schizotypy.

**TABLE 3:** Mean of the median reaction times (milliseconds) to related and unrelated word pairs.

	High AVH prone		Low AVH prone	
	Related	Unrelated	Related	Unrelated
High CP schizotypy	906 (24)	1070 (33)	889 (35)	1103 (48)
Low CP schizotypy	785 (35)	974 (48)	853 (22)	950 (31)

*Standard errors of the mean shown in parentheses.*

#### Additional Analysis

A measure was calculated by subtracting the “related response time” from the “unrelated response time” for each participant. Using correlations we found response times between dominant and subordinate word pairs were significantly positively correlated (High CP schizotypy, High AVH prone [*r*(59) = 0.77, *p* < 0.001]; High CP schizotypy, Low AVH prone [*r*(28) = 0.654, *p* < 0.001]; Low CP schizotypy, High AVH prone [*r*(27) = 0.84, *p* < 0.001]; Low CP schizotypy, Low AVH prone [*r*(68) = 0.656, *p* < 0.001]. Therefore, the reaction time measure is similarly affected by semantic relatedness across the groups, suggesting similar mechanisms are involved in processing the meaning of the word pairs.

### Item Analysis (*F2*) for Reaction Time Data

An analysis with items as cases was used to confirm the results obtained by the previous by-subject (*F1*) analysis. Congruity across *F1* and *F2* analyses indicates true significant differences between groups. If results are not congruent, this may indicate that a few items (or individuals) are driving the differences in reaction time performances. Median reaction times were calculated across participants for every pair of stimuli for each concordant (correct) response. Responses were analyzed in a 2 (meaning) × 2 (relatedness) × 2 (CP schizotypy group) × 2 (AVH proneness group) repeated measures ANOVA. In this analysis CP schizotypy and AVH proneness group became the within-item variables, and meaning and relatedness the between-item variables.

#### Task Effects

There was a significant effect of relatedness [*F*(1, 281) = 37.12, *p* < 0.001, η^2^*_*p*_* = 0.117], which confirms *F1* analysis findings [Related: *M* = 887 ms (S.E. 16), Unrelated: *M* = 1028 ms (S.E. 16)]. A significant effect was also found for meaning [*F*(1, 281) = 14.52, *p* < 0.001, η^2^*_*p*_* = 0.049], with participants responding slower to subordinate word pairs, again supporting the *F1* analysis [Dominant: *M* = 914 ms (S.E. 16), Subordinate: *M* = 1001 ms (S.E. 16)]. An interaction effect was found between meaning and relatedness [*F*(1, 281) = 26.02, *p* < 0.001, η^2^*_*p*_* = 0.085, Dominant-Related: *M* = 785 ms (S.E. 23), Dominant-Unrelated: *M* = 1043 ms (S.E. 23), Subordinate-Related: *M* = 990 ms (S.E. 23), Subordinate-Unrelated: *M* = 1013 ms (S.E. 23)]. Similar to that found in the *F1* analysis, this interaction reflects the task effect where for unrelated words, meaning is not expected to influence responding, as both dominant and subordinate pairs are considered equal in their “unrelatedness.”

#### Group Effects

A significant main effect was found for CP schizotypy group [*F*(1, 281) = 166.18, *p* < 0.001, η^2^*_*p*_* = 0.372) where the high group responded significantly slower compared to the low group (High CP: *M* = 1009 ms (S.E. 13), Low CP: M(low) = 906 ms (S.E. 11)]. CP schizotypy also interacted significantly with meaning [*F*(1, 281) = 4.89, MSE = 0.089, *p* = 0.028, η^2^*_*p*_* = 0.017]. Follow-up analyses using pairwise comparisons revealed that dominant word pairs were responded to slower in the high CP schizotypy group compared to the low group [*F*(1, 141) = 79.51, MSE = 1.04, *p* < 0.001, η^2^*_*p*_* = 0.361, High CP: *M* = 956 ms (S.E. 17), Low CP: *M* = 871 ms (S.E. 15)]. Similarly subordinate word pairs were responded to slower by the high CP group compared to the low group [*F*(1, 140) = 88.63, MSE = 2.06, *p* < 0.001, η^2^*_*p*_* = 0.388, High CP: *M* = 1062 ms (S.E. 20), Low CP: *M* = 941 ms (S.E. 16)]. No significant interactions were found between schizotypy groups and relatedness (*p* = 0.204), or the relatedness and meaning interaction (*p* = 0.899).

The main effect of AVH proneness was significant [*F*(1, 281) = 12.66, MSE = 0.25, *p* < 0.001, η^2^*_*p*_* = 0.043], with the high AVH prone group responding significantly faster to items compared to the low AVH prone group [High AVH: *M* = 943 ms (S.E. 11), Low AVH: *M* = 972 ms (S.E. 13)]. No significant interactions were found between AVH proneness and meaning (*p* = 0.199) or relatedness (*p* = 0.261). The 3-way interaction between AVH proneness, meaning and relatedness was also not significant (*p* = 0.351).

**FIGURE 1:**
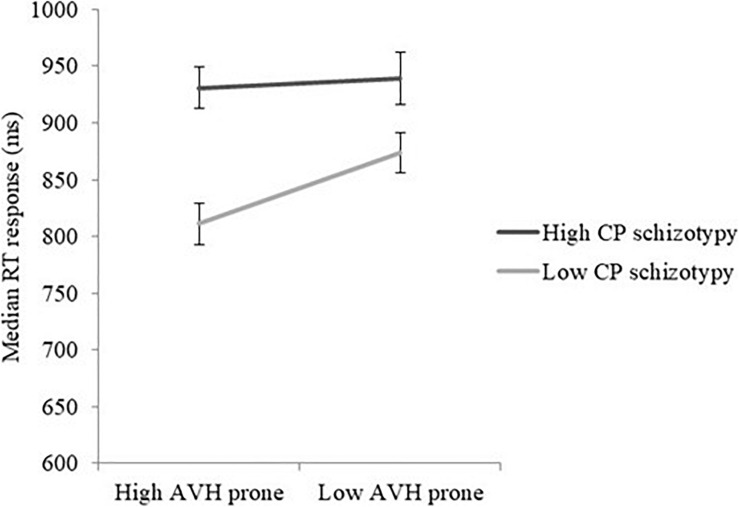
Median reaction time (RT), response (in milliseconds) to relate word pairs. Line indicates Cognitive-Perceptual (CP) schizotypy group with responses broken down according to auditory verbal Hallucination (AVH) proneness.

No interaction was found between CP schizotypy and AVH proneness (*p* = 0.125). The CP schizotypy and AVH proneness interaction effect did not interact with meaning (*p* = 0.224), however it did interact with relatedness [*F*(1, 281) = 5.13, *p* = 0.024, η^2^*_*p*_* = 0.018]. To unpack this interaction the analysis was rerun with the file split by relatedness, which revealed a significant interaction effect between CP schizotypy and AVH proneness in the related condition [*F*(1, 140) = 7.12, *p* = 0.009, η^2^*_*p*_* = 0.048], but not in the unrelated condition (*p* = 0.605). Paired Samples *t*-test indicated that for those in the low CP schizotypy group, responses were significantly faster when combined with high AVH proneness as opposed to low AVH proneness [*t*(142) = -4.54, *p* < 0.0001, Low CP-High LSHS: *M* = 811 ms (S.E. 17), Low CP-Low LSHS: *M* = 873 ms, S.E. = 17, see Figure 1]. Response times in the high CP schizotypy group did not differ as a result of AVH proneness (*p* = 0.667).

#### Consistency of Results Across *F1* and *F2* Analyses

Congruity across *F1* and *F2* analyses is indicative of true differences in CP schizotypy and AVH proneness group effects. The analyses consistently significant across both are:

1. Task effects of meaning, relatedness, and their interaction;
2. Group effects of CP schizotypy, indicating slower response times of those in the high CP schizotypy group;
3. Significant interaction between CP schizotypy, AVH proneness and relatedness, indicating faster responses of the low CP Schizotypy/High AVH prone group to related words.
4. However, the main effect of the high AVH proneness group for response times, and the interaction of CP schizotypy with meaning were only significant in the *F2* analysis and not the *F1* analysis.

### Signal Detection Analyses

Next the data were considered using signal detection analyses (see Table 4).

**TABLE 4:** Mean values for sensitivity and relatedness judgments in Cognitive-Perceptual (CP) schizotypy and Auditory Verbal Hallucination (AVH) prone groups.

Group	Meaning	% Hits	% False alarms	*d′*	*c′*
High CP Schiz,	Dominant	82.7 (14)	13 (10)	2.25 (0.71)	0.03 (0.42)
High AVH prone	Subordinate	62.2 (14)		1.52 (0.54)	
High CP Schiz,	Dominant	84.1 (14)	16.8 (14)	2.13 (0.94)	−0.17 (1.04)
Low AVH prone	Subordinate	62.6 (14)		1.39 (0.67)	
Low CP Schiz,	Dominant	82.9 (16)	12.3 (8)	2.35 (0.78)	−0.22 (1.4)
High AVH prone	Subordinate	63.9 (16)		1.59 (0.59)	
Low CP Schiz,	Dominant	78.2 (19)	14.8 (12)	2.27 (0.96)	0.23 (1.57)
Low AVH prone	Subordinate	57.6 (15)		1.48 (0.67)	

*Hits are out of 36 trials each, and false alarms are out of 72 trials (the sum of both dominant and subordinate conditions). Standard deviation in parentheses. None of the reported differences between groups reached significance at the p < 0.05 level.*

#### Sensitivity Analysis (*d′*)

The sensitivity (*d′*) measure was analyzed in a Repeated Measures ANOVA, with CP schizotypy and AVH proneness the between subject variables, and meaning (dominant or subordinate) the within subject variable. There was a main effect of meaning for dominant and subordinate targets [*F*(1, 178) = 466.333, *p* < 0.001, η^2^*_*p*_* = 0.724], participants significantly discriminated dominant targets (*M* = 2.25, S.E. = 0.07) more easily than subordinate (*M* = 1.5, S.E. = 0.05). There were no significant main effects for CP schizotypy (*p* = 0.402) or AVH proneness (*p* = 0.331). No interaction was observed between CP schizotypy and AVH proneness groups (*p* = 0.907) in the sensitivity analysis.

#### Relative Criterion Analysis (*c′*)

Results indicated that all groups use the same criterion regardless of stimuli type. Mean values were: High CP schizotypy = 1.2 (SD 0.52), Low CP schizotypy = 1.19 (SD 0.47), High AVH prone = 1.25 (SD 0.46), Low AVH prone = 1.16 (SD 0.43).

To compare *c* between CP schizotypy and AVH prone groups *c′* was used (Macmillan and Creelman, 2005). Results indicated that there were no significant main effects for CP schizotypy (*p* = 0.686) or AVH proneness (*p* = 0.544). Additionally, no interaction was found between CP schizotypy and AVH proneness (*p* = 0.093).

## Discussion

The current study considered whether people differed in their performance on a semantic processing task according to their scores on hallucinatory proneness and positive schizotypy. The semantic task primed words according to their dominant or subordinate meaning in an attempt to see whether participants differed in their speed to construe meaning or relations between the words presented. Therefore, participants were divided into groups according to their state hallucinatory predisposition or trait positive schizotypy. This state-trait distinction may be considered debatable by some readers. The LSHS asks people to indicate whether they have had different types of perceptual experiences, moving from vivid imagery through to more fully formed hallucinations (Launay and Slade, 1981). Measures which capture experiences or symptoms are subject to a number of biases in recall (Solhan et al., 2009). People tend to average over their experiences (Kemp et al., 2008), which means that most recent experiences and current state highly influence responses (Gorin and Stone, 2001). Even when experiences can be linked to a specific trigger, such as substance use, it is recognized that concurrent rating is more meaningful than retrospective recall (Mason et al., 2008). This implies that the factors which drive responses to perceptual experience measures are recent, state-like and subject to fluctuations. By contrast, trait-like measures, such as the SPQ, display much more consistency in their responses (Fleeson and Gallagher, 2009; Edmondson et al., 2013; Wang et al., 2018). Similar distinctions for experiences along the psychosis continuum have been made for state and trait anhedonia (Cohen et al., 2011), therefore it is not out of keeping with this area to separate hallucinations from the broader construct of positive schizotypy (Rössler et al., 2013; Unterrassner et al., 2017). Consequently, in the current study we considered the potential interaction and independent effects for AVH proneness, as assessed by the LSHS, and positive schizotypy, captured by CP from the SPQ. The results from this study suggest that CP schizotypy and AVH proneness differ in how they influence the processing of semantic relations, although our findings did not support our initial hypotheses. Across both *F1* and *F2* analyses, the high CP schizotypy reaction time responses were characterized as slower than the low CP schizotypy group. However, the high AVH prone group responded to word pairs faster than the low AVH prone group. In addition, for related word pairs specifically, the low CP schizotypy group responded significantly faster when coupled with high AVH proneness, as opposed to low CP schizotypy and low AVH proneness. No significant differences were found between groups in the sensitivity and criterion.

Results indicated those who were high on CP schizotypy responded significantly slower than those low on CP schizotypy. Contrastingly, some evidence was found for those predisposed to hallucinations to respond to word pairs faster than their respective low scoring counterparts. These findings are indicative of disparities in how state (AVH) and trait (schizotypy) psychosis risk factors influence processing of semantic relations. The slower overall response speed associated with CP schizotypy suggests increased difficulty in the processing of semantic information. It may be that in trait schizotypy, a diffuse spread of semantic activation results in more semantic nodes being activated (Johnston et al., 2008). This increased number of activated associates is hypothesized to result in more time to reach a decision, due to greater difficulty identifying the specific association involved (Neill et al., 2014). Although this diffuse activation results in a slowed response time, it does not appear to compromise accuracy. Support for diffuse, right hemisphere dominated activation, has been reported in relation to positive schizotypy in other studies (Gianotti et al., 2001). The results of our study are consistent with the suggestion that the semantic network in schizotypy may be characterized by a more diffuse spread of activation, which results in a slower response time.

In contrast, the relatedness effects demonstrated by the high AVH prone group in one (but not both) reaction time analyses suggests disinhibited processes may be contributing to significantly faster task completion. In non-clinical AVH samples, the tendency to jump to conclusions and interpret an internally generated experience as a true sensory experience has been suggested as a central mechanism in the generation and maintenance of hallucinations (Brookwell et al., 2013). The current findings contribute tentative support to this mechanism. However, given that this finding was not consistent across both reaction time analyses caution should be made when interpreting this result. Further investigation of reaction time responses to ambiguous semantic relations in AVH proneness is warranted to determine whether these findings are a true effect (Tsakanikos and Reed, 2005; Grant et al., 2014).

Although not predicted, compared to those with low CP schizotypy and low AVH proneness, those with high CP schizotypy and high AVH proneness responded to related word pairs significantly slower, whilst those with low CP schizotypy and high AVH proneness responded to related word pairs significantly faster. This interaction suggests that there may be two mechanisms at work. CP schizotypy appears to result in a more diffuse spread of semantic activation, which slows response times to related word pairs. Contrastingly, AVH proneness seems to reflect disinhibitive processes, such that relationships between semantic associates are responded to significantly faster as long as schizotypy is low/normal. These findings indicate that high CP schizotypy has a far more influential effect on the atypical processing of semantic relations than AVH proneness. These findings suggest that hallucination proneness is separable from positive trait schizotypy. Such a finding is in line with previous research (Paulik et al., 2007; Daalman et al., 2011; de Leede-Smith and Barkus, 2013), and points toward differential trajectories with varying clinical risk.

A priming measure was also calculated for each participant for both dominant and subordinate words and, across all groups, correlations were similar in magnitude. Although the speed of processing differs between groups, the current study suggests that the organization of the semantic system is similar, at least for normatively associated words. These finding suggest that scoring highly on CP schizotypy or AVH proneness has no effect on the ability to detect relationships between words. In addition, no significant differences were found between the CP schizotypy and AVH prone groups for signal detection outcomes. Research suggests high schizotypes require additional task demands before the breakdown in control processes (such as inhibition) that organize semantic processing occurs (Niznikiewicz et al., 2002). Since our task was relatively simple, a low level demand was placed on participants cognitive resources. Further cognitive load may be required before schizotypes adopt a less conservative decision-making style under ambiguous conditions (e.g., Grimshaw et al., 2010).

A number of limitations need to be taken into account. The sample consisted of reasonably high functioning university students who generally have higher cognitive, social, and often financial resources compared to community samples. Consequently the failure to find significant differences in signal detection criteria may be the result of the current sample not being representative of the spread of ability in the general population. However, high error rates on the NART suggest we did have a wide spread of verbal ability. Furthermore, the current study used the CP schizotypy factor to split high and low schizotypal groups. Although this has been used in previous studies testing for semantic processing abnormalities (Niznikiewicz et al., 2002; Johnston et al., 2008; Kostova et al., 2011), larger differences in semantic function are observed when psychosis prone groups are characterized on positive scores on language and thought deviations (Spitzer, 1997). Certainly schizophrenia patients with thought disorder display the greatest aberrations in semantic system functioning (Pomarol-Clotet et al., 2008). Perhaps splitting psychosis prone groups on a language/thought deviation measure would provide more sensitivity for considering semantic relations.

In conclusion, this study considered the nature of semantic processing disturbances in both high trait CP schizotypy and high state AVH prone groups. Our findings indicate that the speed of processing ambiguous semantic relations varies according to level of trait and state psychosis risk. From these initial comparisons, it appears that the slower speed of semantic processing found in high CP schizotypy may be related to a more diffuse spread of semantic activation. Contrastingly the semantic processing capabilities associated with AVH proneness may be related to disinhibitive processes, resulting in an accurate and efficient speed of decision making for semantic information, but only in the context of low CP schizotypy. Previously, positive schizotypy and AVH proneness were believed to be synonymous indications but our study suggests further investigation is required to determine the separation between these two phenotypes.

## Data Availability Statement

The datasets generated for this study are available on request to the corresponding author.

## Ethics Statement

The study was reviewed and approved by the Social Sciences and Humanities Human Research Ethics Committee at the University of Wollongong. The participants provided written informed consent to participate in this study.

## Author Contributions

SL-S was involved in the study design, led the data collection, wrote the first draft of the manuscript, and performed the analysis. SR and EB contributed to the study design, analysis, and were involved in writing the manuscript. LH, SM, and EM were involved in the data collection and proofread and contributed to the manuscript. All authors contributed to the article and approved the submitted version.

## Conflict of Interest

The authors declare that the research was conducted in the absence of any commercial or financial relationships that could be construed as a potential conflict of interest.
